# Phylogenetic estimation of the viral fitness landscape of HIV-1 set-point viral load

**DOI:** 10.1093/ve/veac022

**Published:** 2022-03-16

**Authors:** Lele Zhao, Chris Wymant, François Blanquart, Tanya Golubchik, Astrid Gall, Margreet Bakker, Daniela Bezemer, Matthew Hall, Swee Hoe Ong, Jan Albert, Norbert Bannert, Jacques Fellay, M Kate Grabowski, Barbara Gunsenheimer-Bartmeyer, Huldrych F Günthard, Pia Kivelä, Roger D Kouyos, Oliver Laeyendecker, Laurence Meyer, Kholoud Porter, Ard van Sighem, Marc van der Valk, Ben Berkhout, Paul Kellam, Marion Cornelissen, Peter Reiss, Christophe Fraser, Luca Ferretti

**Affiliations:** Big Data Institute, Li Ka Shing Centre for Health Information and Discovery, Nuffield Department of Medicine, University of Oxford, Old Road Campus, Headington, Oxford OX3 7LF, UK; Big Data Institute, Li Ka Shing Centre for Health Information and Discovery, Nuffield Department of Medicine, University of Oxford, Old Road Campus, Headington, Oxford OX3 7LF, UK; Centre for Interdisciplinary Research in Biology (CIRB), Collège de France, CNRS, INSERM, PSL Research University, Cedex 05, Paris 75231, France; Big Data Institute, Li Ka Shing Centre for Health Information and Discovery, Nuffield Department of Medicine, University of Oxford, Old Road Campus, Headington, Oxford OX3 7LF, UK; European Molecular Biology Laboratory, European Bioinformatics Institute, Wellcome Genome Campus, Cambridge CB10 1SD, UK; Laboratory of Experimental Virology, Department of Medical Microbiology and Infection Prevention, Amsterdam University Medical Centers, University of Amsterdam, Amsterdam, MB 1007, Netherlands; Stichting HIV Monitoring, Amsterdam, Amsterdam, AZ 1105, Netherlands; Big Data Institute, Li Ka Shing Centre for Health Information and Discovery, Nuffield Department of Medicine, University of Oxford, Old Road Campus, Headington, Oxford OX3 7LF, UK; Wellcome Sanger Institute, Wellcome Genome Campus, Hinxton, Cambridge CB10 1SA, UK; Department of Microbiology, Tumor and Cell Biology, Karolinska Institutet, Solna, Stockholm 171 77, Sweden; Department of Clinical Microbiology, Karolinska University Hospital, Solna, Stockholm S-171 76, Sweden; Division for HIV and Other Retroviruses, Department of Infectious Diseases, Robert Koch Institute, Berlin 13353, Germany; School of Life Sciences, Ecole Polytechnique Fédérale de Lausanne, Lausanne CH-1015, Switzerland; Swiss Institute of Bioinformatics, Lausanne 1015, Switzerland; Precision Medicine Unit, Lausanne University Hospital and University of Lausanne, Lausanne CH-1015, Switzerland; Department of Pathology, John Hopkins University, Baltimore, MD 21287, USA; Department of Infectious Disease Epidemiology, Robert Koch Institute, Berlin 13353, Germany; Division of Infectious Diseases and Hospital Epidemiology, University Hospital Zurich, Zurich CH-8091, Switzerland; Institute of Medical Virology, University of Zurich, Zurich 8057, Switzerland; Department of Infectious Diseases, Helsinki University Hospital, Helsinki FI-00029, Finland; Division of Infectious Diseases and Hospital Epidemiology, University Hospital Zurich, Zurich CH-8091, Switzerland; Institute of Medical Virology, University of Zurich, Zurich 8057, Switzerland; Division of Intramural Research, NIAID, NIH, Baltimore, MD 21205, USA; INSERM CESP U1018, Université Paris Saclay, APHP, Service de Santé Publique, Hôpital de Bicêtre, Le Kremlin-Bicêtre 94270, France; Institute for Global Health, University College London, London WC1N 1EH, UK; Stichting HIV Monitoring, Amsterdam, Amsterdam, AZ 1105, Netherlands; Stichting HIV Monitoring, Amsterdam, Amsterdam, AZ 1105, Netherlands; Laboratory of Experimental Virology, Department of Medical Microbiology and Infection Prevention, Amsterdam University Medical Centers, University of Amsterdam, Amsterdam, MB 1007, Netherlands; Kymab Ltd, Babraham Research Campus, Cambridge CB22 3AT, UK; Department of Infectious Diseases, Faculty of Medicine, Imperial College London, South Kensington Campus, London SW7 2AZ, UK; Laboratory of Experimental Virology, Department of Medical Microbiology and Infection Prevention, Amsterdam University Medical Centers, University of Amsterdam, Amsterdam, MB 1007, Netherlands; Molecular Diagnostic Unit, Department of Medical Microbiology and Infection Prevention, Amsterdam University Medical Centers, University of Amsterdam, Amsterdam, MB 1007, Netherlands; Stichting HIV Monitoring, Amsterdam, Amsterdam, AZ 1105, Netherlands; Department of Global Health, Amsterdam University Medical Centers, University of Amsterdam and Amsterdam Institute for Global Health and Development, Amsterdam, DE 1100, Netherlands; Big Data Institute, Li Ka Shing Centre for Health Information and Discovery, Nuffield Department of Medicine, University of Oxford, Old Road Campus, Headington, Oxford OX3 7LF, UK; Big Data Institute, Li Ka Shing Centre for Health Information and Discovery, Nuffield Department of Medicine, University of Oxford, Old Road Campus, Headington, Oxford OX3 7LF, UK

**Keywords:** HIV-1, between-host evolution, tansmission fitness, set-point viral load

## Abstract

Set-point viral load (SPVL), a common measure of human immunodeficiency virus (HIV)-1 virulence, is partially determined by viral genotype. Epidemiological evidence suggests that this viral property has been under stabilising selection, with a typical optimum for the virus between 10^4^ and 10^5^ copies of viral RNA per ml. Here we aimed to detect transmission fitness differences between viruses from individuals with different SPVLs directly from phylogenetic trees inferred from whole-genome sequences. We used the local branching index (LBI) as a proxy for transmission fitness. We found that LBI is more sensitive to differences in infectiousness than to differences in the duration of the infectious state. By analysing subtype-B samples from the Bridging the Evolution and Epidemiology of HIV in Europe project, we inferred a significant positive relationship between SPVL and LBI up to approximately 10^5^ copies/ml, with some evidence for a peak around this value of SPVL. This is evidence of selection against low values of SPVL in HIV-1 subtype-B strains, likely related to lower infectiousness, and perhaps a peak in the transmission fitness in the expected range of SPVL. The less prominent signatures of selection against higher SPVL could be explained by an inherent limit of the method or the deployment of antiretroviral therapy.

## 1. Introduction

At the end of 2020, an estimated 38 million people worldwide were living with human immunodeficiency virus (HIV), with roughly 73 per cent of these individuals accessing antiretroviral therapy (ART; www.unaids.org). For many, HIV has become a manageable chronic condition, thanks to the treatment becoming increasingly accessible through healthcare policies and infrastructural developments. However, much work remains to end the acquired immune deficiency syndrome (AIDS) epidemic as a global health threat. Ongoing transmission in under-surveyed key populations still feeds into the epidemic (Nduva et al. 2020). Therefore, understanding the risk factors for transmission, including viral genetic ones, is paramount for the control and mitigation of the epidemic.

Untreated HIV infections progress in three stages: acute, chronic, and AIDS stage (Alizon et al. 2010). In the chronic stage, a relatively stable viral count per unit volume of plasma blood is established in the viral host (de Wolf et al. 1997). Viral load gradually increases during the chronic stage (O’Brien et al. 1998; Sabin et al. 2000) and accelerates during the final stage of AIDS. The relatively stable viral load over the chronic stage is termed set-point viral load (SPVL), which varies between individuals from 10^2^ to 10^6^ copies/ml (Bonhoeffer et al. 2003; Fraser et al. 2014). SPVL has been observed to correlate with disease progression in the absence of treatment, with higher viral loads leading to more rapid progression to AIDS (Fraser et al. 2007, 2014). While host genetics (e.g. human leukocyte antigen (HLA) system) and other environmental factors contribute to the variation of SPVL between individuals, viral genetics have been found to account for a third of the heritability of SPVL with data from both European (Blanquart et al. 2017; Bertels et al. 2018; Mitov and Stadler 2018) and sub-Saharan African cohorts (Hollingsworth et al. 2010; Lingappa et al. 2013; Yue et al. 2013).

SPVL is a proxy for the amount of circulating virus within an infected individual and therefore determines the potential infectiousness of the individual. SPVL can also be viewed as a reflection of the burden imposed on an infected individual’s immune system, which affects the duration of the chronic stage of the infection in the absence of treatment. During the chronic stage, the combined effect of infectiousness and duration of infectiousness on the average number of transmissions can be summarised in a single-peaked landscape for the transmission potential as a function of SPVL (Fraser et al. 2007). Surveillance data showed an approximately lognormal distribution for SPVL, with the most common values being those expected to have the greatest transmission potential, suggesting a selective process acting to balance infectiousness and duration of chronic stage to maximise transmission potential. By comparing viral loads of infected individuals found to be in transmission clusters against non-cluster members, Wertheim et al. showed that higher viral loads at diagnosis are selected among HIV transmission networks in the USA (Wertheim et al. 2019).

In classical epidemiological terms, the transmission potential of a pathogen is related to its basic reproductive number within a given population. With genetic data and phylogenetic methods, the transmission potential can be calculated in different ways (Faria et al. 2018). Phylogenetic trees are commonly used to reconstruct relationships between sequences from samples of pathogens in calendar time or molecular time. Signals extracted from phylogenies have proven useful in inferring the fitness of the sampled organisms (Neher, Russell, and Shraiman 2014). Specifically, fitness estimated from phylogenies of sequences across time can inform evolutionary trajectories of genotypes present in an asexual pathogen population, as demonstrated in retrospective predictions of circulating influenza A/H3N2 strains (Neher, Russell, and Shraiman 2014). One method developed to achieve this is the local branching index (LBI), which calculates the integrated exponentially discounted tree length surrounding a focal node/tip with a timescale parameter (*τ*) denoting the tree neighbourhood within which fitness is ‘remembered’ (Neher, Russell, and Shraiman 2014). In other words, the LBI will be larger at a focal node/tip when the tree branches more frequently near this node/tip, and a larger rate of branching reflects a higher transmission fitness of the viral genotype represented by the focal node/tip compared to other parts of the tree.

In this study, using LBI, we demonstrated the signal of selection on transmission fitness among HIV-1 subtype-B genotypes from individuals with different SPVLs. Powered by a large sample size from the Bridging the Evolution and Epidemiology of HIV in Europe (BEEHIVE) project and the stringent selection criteria in procuring these samples (Blanquart et al. 2017), we show that transmission fitness varies with SPVL (*P*-value = 0.009), with our central estimate for the variation being an increase until a peak of approximately 10^5^ copies/ml. This relationship holds true for all sliding windows across the HIV-1 genome, except for the last 1,500 bp. The relationship between transmission fitness and SPVL higher than ∼10^5^ copies/ml is confounded by methodological and epidemiological factors. Interpreting LBI in terms of a viral birth–death epidemic/phylodynamic model, we show that variation in LBI is more sensitive to variation in lineage birth rate (infectiousness) at shorter timescales, while accounting for variation in lineage death rates (duration of infectious state) at longer timescales.

## 2. Methods

### 2.1 Data

The data set we analysed consists of *N* = 1,927 whole-genome subtype-B consensus sequences from the BEEHIVE project, collected across European cohorts. Viral RNA was manually extracted from samples (Cornelissen et al. 2017) and then reverse transcribed and amplified using primers to define four overlapping amplicons spanning the whole genome, which were fragmented and sequenced with Illumina MiSeq or HiSeq platforms (Gall et al. 2012). Whole-genome consensus sequences were reconstructed from the resulting short-read data using Iterative Virus Assembler (Hunt et al. 2015) and shiver (Wymant et al. 2018). Sequence subtypes were determined using the COMET software (Struck et al. 2014) and validated using phylogenetic tree placement methods (Blanquart et al. 2017). SPVLs were single measurements of viral load performed on the same sample used for sequencing, with samples taken between 6 and 24 months after seroconversion. This measure of viral load during the set-point window of infection was previously shown to have a greater heritability than an average of multiple measurements taken at different times (Blanquart et al. 2017).

To avoid the uneven representation of the epidemic caused by inconsistent sampling, we restricted our regression analysis data set to contain only samples from the period 2000–12 (see Supplement Figure 1), during which the sampling distribution was relatively constant. We also excluded samples with viral load measured as less than 1,000 copies/ml, given that there are biologically induced experimental limits to accurate reporting and detection of low viral load samples. This filter not only increased our confidence in the SPVL measurements used in the regression, but also removed the potential bias on LBI estimations caused by lower genomic coverage for low viral load samples, which may potentially lead to noisier measurement of the viral genotype and therefore longer terminal branches.

### 2.2 Phylogenetic inference

We created a multiple-sequence alignment by merging individual pairwise alignments between each sample’s whole-genome consensus sequence and the HXB2 reference genome. We also took sliding-window subsets of this whole-genome alignment, with a width of approximately 1,000 bp with ∼500-bp overlap. All alignments were used to build maximum-likelihood trees using IQ-TREE (Nguyen et al. 2015), with the substitution model general time reversible model + F + R4. Time calibration was done using treedater (Volz and Frost 2017), with the year of sampling for each sequence. When the year of sampling was unavailable, we used the year of seroconversion.

### 2.3 Transmission fitness computations

**Figure 1.**
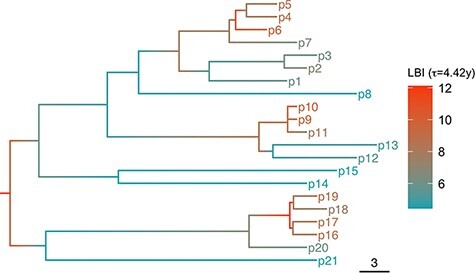
Example tree coloured with LBI values along branches and nodes, showing higher LBI values for more frequently branching regions of the tree.

We computed the LBI (Neher, Russell, and Shraiman 2014) for each tip of the time-calibrated trees, using an approach adapted from Equations 17–19 of Neher, Russell, and Shraiman (2014) (available at github.com/BDI-pathogens/hiv_spvl_fitness/). We used the recommended timescale parameter (*τ*) (Neher, Russell, and Shraiman 2014), namely 0.0625 multiplied by the mean cophenetic distance of tips in the phylogeny, corresponding to *τ* = 4.42 years for the tree in this study. An example tree coloured with LBI values is shown in Fig. 1. We also varied *τ* in sensitivity analyses. To compute a *z*-score for the LBI while accounting for the uneven availability of samples in different years, we permuted the branching patterns of time-calibrated trees 1,000 times while keeping the coalescent events and the year of samples constant (Dearlove and Frost 2015). We used the LBI values from all permuted trees to calculate the *z*-score of the raw LBI values for each tip in the non-permuted tree.

We also converted raw LBI estimates for the subset of *N* = 1,542 samples from men who have sex with men (MSM) into lineage birth and death rates. To infer lineage birth rates $\beta $, we assume a fixed death rate of ${\delta _0} = 0.15/year$ inferred by maximum likelihood from the tree sliced between 2000 and 2012 and then we interpret changes in the LBI as changes in $\beta $ according to the equation for birth–death models (see ‘Interpretation of LBI under a birth–death model’). For a large tree with $\beta - \delta \lt \lt 1/\tau $, the relation can be expressed analytically:

$$\beta = {{1 + \tau {\delta _0}} \over \tau }\left( {1 - {\tau \over {{I_{LB}}}}} \right)$$

where ${I_{LB}}$ denotes the LBI value, $\beta $ is the lineage birth rate, ${\delta _0} $ is the lineage death rate, and $\tau $ is the LBI timescale parameter. Here however we solve numerically the exact relation (see Supplementary File 1) assuming a finite tree with root in 1995 and tips until 2012. In principle, lineage death rates $\delta $ may be inferred similarly by assuming a fixed birth rate of ${\beta _0} = 0.14/year$ also inferred by maximum likelihood and then interpreting changes in LBI as changes in $\delta $, but their inference is noisier and more prone to biases, as well as less relevant for the results in this paper.

### 2.4 Regression of LBI and SPVL

Gaussian process regressions—a non-parametric regression/smoothing approach based on a Gaussian process prior—were applied to normalised quantile-transformed *z*-scores of LBI estimations and corresponding quantile-transformed SPVL measurements. The squared exponential covariance function was used:

$$\Sigma = k\left( {{x_i},{x_j}} \right) = {\sigma ^2}exp\left( { - {{{{\left( {{x_i} - {x_j}} \right)}^2}} \over {2{l^2}}}} \right)$$

with length parameter (${l^2}$) 0.15, variance in data (${\sigma ^2}$) 0.5, and variance in noise 0.5. A permutation test was used to assess the significance of the existence of some relationship between LBI and SPVL: the variance of the regression curve calculated from the true data was compared with the right tail of the distribution of variances of the regression curves calculated from 1,000 permutations of the normalised quantile-transformed *z*-score of LBI values. This defines the *P*-value for the null hypothesis, namely that LBI is independent of SPVL.

## 3. Results

### 3.1 Relationship between transmission potential and SPVL

**Figure 2.**
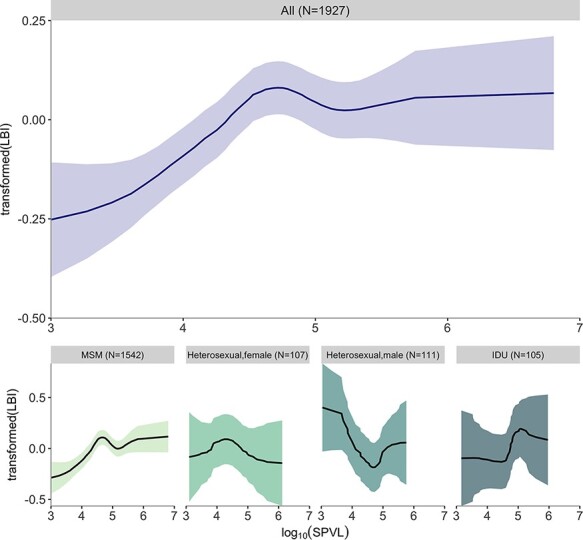
Relationship of transformed LBI and log_10_ SPVL for all samples and subsets of different modes of transmission and sex. Solid lines and shading are posterior mean and 95 per cent confidence interval. Subsets include MSM, heterosexual females, heterosexual males, and IDUs. The full data set and the subset of MSM samples both showed a significant relationship between transformed LBI and SPVL.

We applied Gaussian process regression to our proxy of transmission fitness (i.e. normalised quantile-transformed *z*-score of LBI values) and quantile-transformed SPVL measurements. The regression trend showed a clear increase in transmission fitness with increasing SPVL until approximately 10^5^ copies/ml. As SPVL increases beyond 10^5^ copies/mL, the results showed some evidence for a peak and a decline in transmission fitness and then remained constant over the largest SPVL values (Fig. 2). The existence of a non-constant relation between LBI and SPVL is significant by permutation (*P* = 0.009) (Supplementary Figures 2–3). We further investigated if LBI differed by transmission mode and sex. The subsets of samples for heterosexual males (*N* = 111), heterosexual females (*N* = 107), and injecting drug users (IDUs; *N* = 105) did not produce significant trends, probably due to limited statistical power (Fig. 2). *N* = 1,542 samples were from MSM; these made up the majority of the full data set (*N* = 1,927), and the relationship between SPVL and LBI for this subset of samples was unsurprisingly similar to that of the full data set (*P* = 0.001).

**Figure 3.**
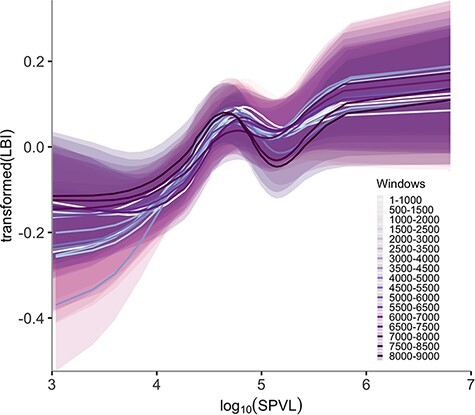
Trends of transformed LBI and log_10_ SPVL estimated from sliding-window alignment built phylogenies across the HIV-1 B genome (note that window widths and coordinates are approximate). Lines are posterior mean values. Shadings are 95 per cent confidence intervals.

To test if the relationship holds true throughout the genome, we divided the genome into 17 overlapping windows roughly 1,000 bases wide. For each window we inferred a phylogeny, calculated transmission fitness at the tips, and estimated the relationship of transmission fitness against SPVL. All windows except the last two (*P* = 0.12 and *P* = 0.18) showed agreement with the global trend (*P* < 0.048) (Fig. 3). The region that did not show significant signatures of the relationship (last 1,500 bp) includes partial sequences of *tat, rev*, and *gp41* genes and the entire *nef* gene. Viral replicative fitness is a direct contributing factor to transmission fitness of HIV-1. While mutations in *gag*, *pol*, and *env* genes directly affect viral replicative fitness, changes in *nef* do so less (Claiborne et al. 2015, reviewed in: Biesinger and Kimata 2008). Therefore, *nef* may be under less selective pressure for enhancing transmission fitness.

### 3.2 Interpretation of LBI under a birth–death model

The LBI at a given point in a tree (such as a tip) is calculated as the length of the whole tree, exponentially discounting contributions with increasing distance from that point. The LBI timescale *τ* corresponds to the size of the relevant surrounding tree neighbourhood (i.e. 1/*τ* is the rate at which distance is discounted). Under a birth–death model for a well-sampled, slowly varying epidemic ($\beta - \delta \lt \lt 1/\tau $), the expected LBI ${I_{LB}}$ is related to the lineage birth rate *β* and the lineage death rate *δ* by the following relation:

$${I_{LB}} = {{1 + } \over {1/\tau - \left( {\beta - \delta } \right)}}$$.

This result is not valid for a fast-growing epidemic nor if the intensity of sampling changes with time; see a detailed derivation in Supplementary File 1 for these scenarios.

**Figure 4.**
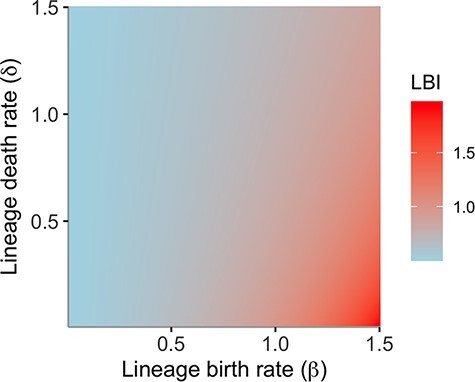
Effect of local lineage birth and death rates on LBI.

In comparison to the conventional transmission potential or basic reproductive number (*R*_0_), we show that LBI is unequally affected by the lineage birth and death rates. Since LBI is related to tree branching processes, it is more sensitive to lineage birth than to lineage death as shown in Fig. 4. Specifically, the difference in LBI between two tips differing in lineage birth rate by $\Delta \beta $ and differing in lineage death rate by $\Delta \delta $ is approximately:

$$\Delta {I_{LB}} = {{I_{LB}^2} \over {1 + \tau \delta }}\left[ {\Delta \beta - \left( {1 - {\tau \over {{I_{LB}}}}} \right)\Delta \delta } \right] = {{I_{LB}^2} \over {1 + \tau \delta }}\left[ {\Delta \beta - {{\tau \beta } \over {1 + \tau \delta }}\Delta \delta } \right]{\rm{ }}.$$

The contribution of the variation in *δ* is suppressed compared to the contribution of the variation in *β*, especially for large values of LBI. In other words, while LBI is one measure of fitness, it is more sensitive to the infectiousness and less sensitive to the duration of the infectious state 1/*δ*. This can be contrasted with other measures of fitness, such as the growth rate $r = \beta - \delta $ or the transmission potential $TP = \beta /\delta $, which are more balanced in their sensitivity to infectiousness and duration of infectiousness. The larger the parameter *τ* is, the stronger the correlation between the difference in LBI between two lineages and the difference in their transmission potential, since $\Delta TP = {1 \over \delta }\left[ {\Delta \beta - {\beta \over \delta }\Delta \delta } \right]$.

**Figure 5.**
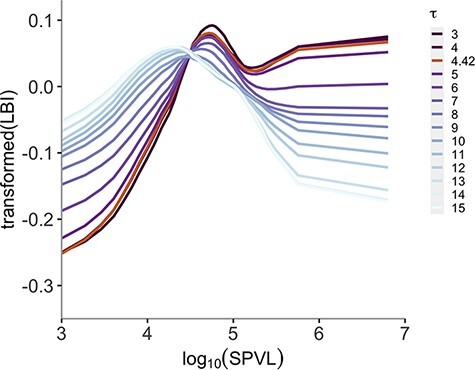
Relationship between transformed LBI and log_10_ SPVL fitted to different LBI timescales (*τ*). Lines are posterior means of each fitting, with gradually lighter colours as *τ* increases.

In order to test the sensitivity of LBI to the lineage death rate, we varied the timescale parameter *τ* from 3 to 15 years. An increasing proportion of lineage deaths is taken into account by LBI estimations when *τ* is larger, thus enhancing sensitivity to the lineage death rate. In fact, we observe the effect of finite lifespan of lineages inside the phylogeny, i.e. the duration of the infectious state, on the curves (Fig. 5). Although these relationships are only significant for *τ* ≤ 5 years (*P* = 0.001–0.019) for our data set, a downward bending trend for higher viral loads started to appear as the value of *τ* increased. This is consistent with the expected shape of the fitness landscape for SPVL and to the idea that different values of *τ* are sensitive to different factors relating to transmission fitness and SPVL. In the context of HIV-1 subtype-B phylogenies, LBI estimates are a proxy for infectiousness (i.e. lineage birth rates) at shorter timescales but capture signatures of selection against high SPVL (i.e. higher lineage death rates) at longer timescales.

### 3.3 Lineage birth rates among samples from MSM

**Figure 6.**
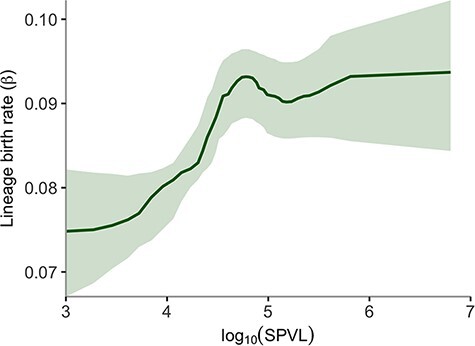
Relationship of lineage birth rate and log_10_ SPVL among samples from MSM. Line and shading are the posterior mean and 95 per cent confidence interval.

For the subset of MSM, we translated the LBI into lineage birth rates using the relationships mentioned in ‘Methods’, showing the result as a function of SPVL in Fig. 6. The difference in lineage birth rate between intermediate SPVL = 10^4.75^ and low SPVL = 10^3^ is approximately 0.0184 per year [0.007–0.029]. Interpreting selection against low SPVL as a result of selection against low infectiousness, this implies an approximate selection coefficient per generation around $s \approx - \Delta \beta /\beta \approx - 0.2$ against low SPVL.

## 4. Discussion

We demonstrated that phylogenetic signals can be used to infer transmission fitness of subtype-B HIV-1 viral genotypes and provided the first direct evidence of a relationship (*P* = 0.009, permutation test) between transmission fitness and SPVL. Transmission fitness is positively correlated with SPVL, until a peak value between 10^4^ and 10^5^ copies/ml. At values of SPVL greater than this peak, transmission fitness may decline, although this is only weakly supported by our results, partly due to a lack of samples with high SPVL and difficulties inherent to the method to detect differences in death rates. In Fraser et al. (2007), the transmission potential, defined as the expected number of people one infected individual could infect during the chronic stage of infection, is the product of transmission rate or infectiousness and the duration of infectious period. For HIV-1 subtype B, the transmission potential is relatively low for individuals with low and high SPVL and high for individual cases with intermediate SPVL. Our application of LBI reproduced this pattern using phylogenetic information, showing selection against low SPVL and possible stabilising selection for SPVL around 10^4^–10^5^ copies of viral RNA/ml. One study concluded that the US epidemic is selecting for high viral loads, which are becoming more common over time (Wertheim et al. 2019). Yet we have seen a decline in viral load (Supplementary Figure 4) for our period of study in European samples, which was also reported in other publications (Pantazis et al. 2014). There could be key differences in what is being selected in these two regional epidemics.

Both theory and analysis demonstrate that LBI can be used to estimate transmission fitness using phylogenetic relationships within a tree. The main assumption for this approach is a panmictic population, without well-defined subpopulations with different coalescent rates. This assumption is not strictly respected for HIV in Europe, but it should not affect our analysis because the dependence of LBI on SPVL should be similar across countries. However, the suggested value for the LBI timescale parameter *τ* for our subtype-B tree (4.42 years) effectively constrains the signal of transmission fitness to this time span before and after the sample was taken, and accounting for exponentially less extending to the future or into the past. Although we do not know the decaying time for transmission fitness for particular HIV genotypes, the timescale parameter is much smaller than the typical duration of HIV infections and therefore constrains the sensitivity of LBI inference of selection on duration of infectious state. In fact, about half of individuals living with HIV in Europe are late presenters (Late presenters working group in COHERE in EuroCoord et al. 2015) who usually live with the infection for more than 4.42 years; therefore, LBI calculations considered a smaller proportion of lineage death events of sampled individuals from the branching patterns of the tree.

In order to estimate relative transmission fitness, LBI implicitly assumes the data captured lineage births and deaths within the specified time period (i.e. a large *τ*), while in HIV infections and transmissions this is not true. Interpreting LBI in the framework of birth–death models, it is more sensitive to relative changes in birth rate or infectiousness in HIV than death rate, at least for death rates <1/*τ*, while transmission potential is affected by both equally. When we increase the value of timescale *τ* used for LBI, relative differences in LBI become a better proxy for relative differences in transmission potential, as the contribution of increased lineage death rates starts to appear by the gradual drop of transmission potential with higher viral load samples. The timescale parameter *τ* may also change the shape of the regression curve for other reasons. It might magnify the variance in estimations for the relatively low number of samples for high SPVL, causing a noticeable change. Also, the increase in *τ* means the focal point of LBI calculations shift from evolutionary history close to the tip in the tree (the sample) to taking into account a longer time span further away from the tip. This effect may depend on how long transmission fitness is maintained by the viral genome through time, which in turn depends on the viral genome’s opportunity to generate new mutations and the interplay between genomic epistasis.

The trends we observed agree with previous modelling results, which found that the transmission potential of HIV-1 subtype B is lower for both low and high SPVLs and highest at intermediate viral loads. However, these estimates were based upon data from untreated individuals (Fraser et al. 2007), and with the current data set, treatment must be considered. If we approximate the effect of ART as reducing infectiousness to zero from the moment of diagnosis and assume the time of diagnosis is before AIDS and independent of SPVL, then the average duration of the infectious state will be independent of SPVL. Transmission potential, which is the product of infectiousness and the duration of the infectious state, is then governed by infectiousness alone. The BEEHIVE data set is a combined European cohorts data set, where ART coverage is above 70 per cent for the sampling years of our data points (CASCADE ∼100 per cent (Stirrup et al. 2018), Netherlands HIV monitoring annual reports 78.3–85 per cent (Stichting HIV Monitoring Annual Report 2003–2012), and Swiss HIV Cohort Study 70–90 per cent (SHCS 2021)). For BEEHIVE, only individuals with samples obtained soon after a known time of seroconversion are included, to avoid biases related to their stage of infection. However, time to ART initiation for individuals in the European cohorts varied from immediately to several years post seroconversion. Variation in the time from infection to ART can be decomposed into variation in the time from infection to diagnosis (Wolbers et al. 2008; van Sighem et al. 2015) and variation in the time from diagnosis to treatment (Boender et al. 2018). During our study period ART guidelines changed, including a CD4 count threshold change around 2008 (Wilkin and Gulick 2008), and only since 2019 has immediate ART been recommended for people living with HIV in Europe (EACS 2019). With a high proportion of individuals on ART and the variation in treatment start time, individuals within the population have variable duration of infectious state, and this can change the relationship between transmission potential and high SPVL (Supplementary Figure 5 and method in Supplementary File 2). Early access to ART will also confound the relationship between the transmission potential and SPVL by increasing the proportion of transmissions during the acute phase of the infection. Before a policy of immediate ART initiation upon entry to care, individuals with higher viral load have a faster progression to the treatment initiation (Mellors et al. 1997), and thus these viral strains are more likely to be removed from the transmission network than those of individuals with low viral load. Clearly, further detailed modelling is needed to characterise and infer the effects of treatment on the landscape of HIV-1 transmission potential.

Many characteristics of individual HIV cases contribute to the transmission potential of the virus, and some of these links may be obscure or highly heterogeneous; therefore, it is important we identify a proficient candidate characteristic to infer transmission potential. By knowing which genotypes have a higher transmission potential, better epidemic preparedness can be achieved. Our study took a step back and inferred general transmission fitness patterns made possible with a large data set from the European HIV cohorts and demonstrated a practical application of LBI to detect selection on a continuous phenotype. The relationship we observed between LBI and SPVL enabled a better understanding of evolutionary selection pressure on transmission fitness. The application of this phylogeny-based method should also be expanded to other viruses and other phenotypes as well, not limited to SPVL.

## Supplementary Material

veac022_Supp

## Data Availability

The tree used for the analyses is available in newick format at github.com/BDI-pathogens/hiv_spvl_fitness.

## References

[R1] Alizon S. et al. (2010) ‘Phylogenetic Approach Reveals That Virus Genotype Largely Determines HIV Set-Point Viral Load’, *PLoS Pathogens*, 6: e1001123. 10.1371/journal.ppat.1001123 PMC2947993 20941398

[R2] Bertels F. et al. (2018) ‘Dissecting HIV Virulence: Heritability of Setpoint Viral Load, CD4+ T-Cell Decline, and Per-Parasite Pathogenicity’, *Molecular Biology and Evolution*, 35: 27–37. 29029206 10.1093/molbev/msx246 PMC5850767

[R3] Biesinger T. , and Kimata J. T. (2008) ‘HIV-1 Transmission, Replication Fitness and Disease Progression’, *Virology: Research and Treatment*, 2008: 49–63. 20354593 PMC2846839

[R4] Blanquart F. et al. (2017) ‘Viral Genetic Variation Accounts for a Third of Variability in HIV-1 Set-Point Viral Load in Europe’, *PLoS Biology*, 15: e2001855.doi: 10.1371/journal.pbio.2001855. PMC5467800 28604782

[R5] Boender T. S. et al. ATHENA national observational HIV cohort . (2018) ‘AIDS Therapy Evaluation in the Netherlands (ATHENA) National Observational HIV Cohort: Cohort Profile’, *BMJ Open*, 8: e022516. 10.1136/bmjopen-2018-022516 PMC6169757 30249631

[R6] Bonhoeffer S. et al. (2003) ‘Glancing behind Virus Load Variation in HIV-1 Infection’, *Trends in Microbiology*, 11: 499–504. 14607066 10.1016/j.tim.2003.09.002

[R7] Claiborne D. T. et al. (2015) ‘Replicative Fitness of Transmitted HIV-1 Drives Acute Immune Activation, Proviral Load in Memory CD4+ T Cells, and Disease Progression’, *Proceedings of the National Academy of Sciences of the United States of America*, 112: E1480–89. 25730868 10.1073/pnas.1421607112 PMC4378387

[R8] Cornelissen M. et al. (2017) ‘From Clinical Sample to Complete Genome: Comparing Methods for the Extraction of HIV-1 RNA for High-Throughput Deep Sequencing’, *Virus Research*, 239: 10–6. 27497916 10.1016/j.virusres.2016.08.004

[R9] de Wolf F. et al. (1997) ‘AIDS Prognosis Based on HIV-1 RNA, CD4+ T-Cell Count and Function: Markers with Reciprocal Predictive Value over Time after Seroconversion’, *AIDS*, 11: 1799–806. 9412697 10.1097/00002030-199715000-00003

[R10] Dearlove B. L. , and Frost S. D. W. (2015) ‘Measuring Asymmetry in Time-Stamped Phylogenies’, *PLoS Computational Biology*, 11: e1004312. 10.1371/journal.pcbi.1004312 PMC4492995 26147205

[R11] EACS . (2019), *European AIDS Clinical**Society Guidelines Version 10.0*. <https://www.eacsociety.org/media/2019_guidelines-10.0_final.pdf> accessed 10 Feb 2022.

[R12] Faria N. R. et al. (2018) ‘Genomic and Epidemiological Monitoring of Yellow Fever Virus Transmission Potential’, *Science*, 361: 894–9. 30139911 10.1126/science.aat7115 PMC6874500

[R13] Fraser C. et al. (2014) ‘Virulence and Pathogenesis of HIV-1 Infection: An Evolutionary Perspective’, *Science*, 343: 1243727. 10.1126/science.1243727 PMC5034889 24653038

[R14] Fraser C. T. et al. (2007) ‘Variation in HIV-1 Set-Point Viral Load: Epidemiological Analysis and an Evolutionary Hypothesis’, *Proceedings of the National Academy of Sciences of the United States of America*, 104: 17441–6. 17954909 10.1073/pnas.0708559104 PMC2077275

[R15] Gall A. et al. (2012) ‘Universal Amplification, next-Generation Sequencing, and Assembly of HIV-1 Genomes’, *Journal of Clinical Microbiology*, 50: 3838–44. 22993180 10.1128/JCM.01516-12 PMC3502977

[R16] Hollingsworth T. D. et al. (2010) ‘HIV-1 Transmitting Couples Have Similar Viral Load Set-Points in Rakai, Uganda’, *PLoS Pathogens*, 6: e1000876. 10.1371/journal.ppat.1000876 PMC2865511 20463808

[R17] Hunt M. et al. (2015) ‘IVA: Accurate de Novo Assembly of RNA Virus Genomes’, *Bioinformatics*, 31: 2374–6. 25725497 10.1093/bioinformatics/btv120 PMC4495290

[R18] Lingappa J. R. et al. (2013) ‘Partner Characteristics Predicting HIV-1 Set Point in Sexually Acquired HIV-1 among African Seroconverters’, *AIDS Research and Human Retroviruses*, 29: 164–71. 23061422 10.1089/aid.2012.0206 PMC3537302

[R19] Mellors J. W. et al. (1997) ‘Plasma Viral Load and CD4+ Lymphocytes as Prognostic Markers of HIV-1 Infection’, *Annals of Internal Medicine*, 126: 946–54. 9182471 10.7326/0003-4819-126-12-199706150-00003

[R20] Mitov V. , and Stadler T. (2018) ‘A Practical Guide to Estimating the Heritability of Pathogen Traits’, *Molecular Biology and Evolution*, 35: 756–72. 29329426 10.1093/molbev/msx328 PMC5850476

[R21] Late presenters working group in COHERE in EuroCoord. Mocroft A. et al. (2015) ‘Late Presentation for HIV Care across Europe: Update from the Collaboration of Observational HIV Epidemiological Research Europe (COHERE) Study, 2010 to 2013’, *Eurosurveillance*, 20: 30070.doi: 10.2807/1560-7917.ES.2015.20.47.30070. 26624933

[R22] Nduva G. M. et al. (2020) ‘HIV-1 Transmission Patterns within and between Risk Groups in Coastal Kenya’, *Scientific Reports*, 10: 6775. 10.1038/s41598-020-63731-z PMC7174422 32317722

[R23] Neher R. A. , Russell C. A., and Shraiman B. I. (2014) ‘Predicting Evolution from the Shape of Genealogical Trees’, *eLife*, 3: e03568.doi: 10.7554/eLife.03568. PMC4227306 25385532

[R24] Nguyen L.-T. et al. (2015) ‘IQ-TREE: A Fast and Effective Stochastic Algorithm for Estimating Maximum-Likelihood Phylogenies’, *Molecular Biology and Evolution*, 32: 268–74. 25371430 10.1093/molbev/msu300 PMC4271533

[R25] O’Brien T. R. et al. (1998) ‘Longitudinal HIV-1 RNA Levels in a Cohort of Homosexual Men’, *Journal of Acquired Immune Deficiency Syndromes and Human Retrovirology: Official Publication of the International Retrovirology Association*, 18: 155–61. 10.1097/00042560-199806010-00007 9637580

[R26] Pantazis N. et al. (2014) ‘Temporal Trends in Prognostic Markers of HIV-1 Virulence and Transmissibility: An Observational Cohort Study’, *The Lancet HIV*, 1: e119–26. 26424120 10.1016/S2352-3018(14)00002-2

[R27] Sabin C. A. et al. (2000) ‘Course of Viral Load Throughout HIV-1 Infection’, *Journal of Acquired Immune Deficiency Syndromes*, 23: 172–7. 10737432 10.1097/00126334-200002010-00009

[R28] Stichting HIV Monitoring . (2003–2012), *Annual Report 2003**–**2012. Human Immunodeficiency Virus (HIV) Infection in the Netherlands*. Amsterdam: Stichting HIV Monitoring, 2003–2012. <www.hiv-monitoring.nl> accessed 7 Feb 2022.

[R29] Stirrup O. T. et al. (2018) ‘Predictors of CD 4 Cell Recovery following Initiation of Antiretroviral Therapy among HIV‐1 Positive Patients with Well‐estimated Dates of Seroconversion’, *HIV Medicine*, 19: 184–94. 29230953 10.1111/hiv.12567 PMC5836945

[R30] Struck D. et al. (2014) ‘COMET: Adaptive Context-Based Modeling for Ultrafast HIV-1 Subtype Identification’, *Nucleic Acids Research*, 42: e144. 10.1093/nar/gku739 PMC4191385 25120265

[R31] Swiss HIV Cohort Study . (2021), SHCS Key Data Current Status. <https://www.shcs.ch/232-current-status> accessed 7 Feb 2022.

[R32] van Sighem A. et al. (2015) ‘Estimating HIV Incidence, Time to Diagnosis, and the Undiagnosed HIV Epidemic Using Routine Surveillance Data’, *Epidemiology*, 26: 653–60. 26214334 10.1097/EDE.0000000000000324 PMC4521901

[R33] Volz E. M. , and Frost S. D. W. (2017) ‘Scalable Relaxed Clock Phylogenetic Dating’, *Virus Evolution*, 3: vex025.doi: 10.1093/ve/vex025.

[R34] Wertheim J. O. et al. (2019) ‘Natural Selection Favoring More Transmissible HIV Detected in United States Molecular Transmission Network’, *Nature Communications*, 10: 5788. 10.1038/s41467-019-13723-z PMC6923435 31857582

[R35] Wilkin T. J. , and Gulick R. M. (2008) ‘When to Start Antiretroviral Therapy?’ *Clinical Infectious Diseases: An Official Publication of the Infectious Diseases Society of America*, 47: 1580–6. 18990069 10.1086/593311

[R36] Wolbers M. et al. (2008) ‘Delayed Diagnosis of HIV Infection and Late Initiation of Antiretroviral Therapy in the Swiss HIV Cohort Study’, *HIV Medicine*, 9: 397–405. 18410354 10.1111/j.1468-1293.2008.00566.x

[R37] Wymant C. et al. (2018) ‘Easy and Accurate Reconstruction of Whole HIV Genomes from Short-Read Sequence Data with Shiver’, *Virus Evolution*, 4: vey007.doi: 10.1093/ve/vey007. PMC5961307 29876136

[R38] Yue L. et al. (2013) ‘Cumulative Impact of Host and Viral Factors on HIV-1 Viral-Load Control during Early Infection’, *Journal of Virology*, 87: 708–15. 23115285 10.1128/JVI.02118-12 PMC3554094

