# Correction to: Phylogenetic estimation of the viral fitness landscape of HIV-1 set-point viral load

**DOI:** 10.1093/ve/veaf080

**Published:** 2025-10-08

This is a correction to: Lele Zhao, Chris Wymant, François Blanquart, Tanya Golubchik, Astrid Gall, Margreet Bakker, Daniela Bezemer, Matthew Hall, Swee Hoe Ong, Jan Albert, Norbert Bannert, Jacques Fellay, M Kate Grabowski, Barbara Gunsenheimer-Bartmeyer, Huldrych F Günthard, Pia Kivelä, Roger D Kouyos, Oliver Laeyendecker, Laurence Meyer, Kholoud Porter, Ard van Sighem, Marc van der Valk, Ben Berkhout, Paul Kellam, Marion Cornelissen, Peter Reiss, Christophe Fraser, Luca Ferretti, on behalf of the BEEHIVE Collaboration, Phylogenetic estimation of the viral fitness landscape of HIV-1 set-point viral load, *Virus Evolution*, Volume 8, Issue 1, 2022, https://doi.org/10.1093/ve/veac022

In the originally published online version of this manuscript, the equation in the first paragraph of section 3.2 Interpretation of LBI under a birth–death model was lacking a term (tau delta). The equation should read:


\begin{align*} {I_{LB}} = {{1 + \tau \delta} \over {1/\tau - \left( {\beta - \delta } \right)}} \end{align*}

The error has been outlined only in this correction notice to preserve the version of record.

