# Supplementary material for: Phylogenetic estimation of the viral fitness landscape of HIV-1 set-point viral load
Source: Virus Evol. 2022 Mar 16;8(1):veac022. doi: 10.1093/ve/veac022 (PMC8986633; doi:10.1093/ve/veac022)
Supplement: veac022_Supp [file veac022_supp.zip › Supplementary Figures.pdf]

Supplementary Figures

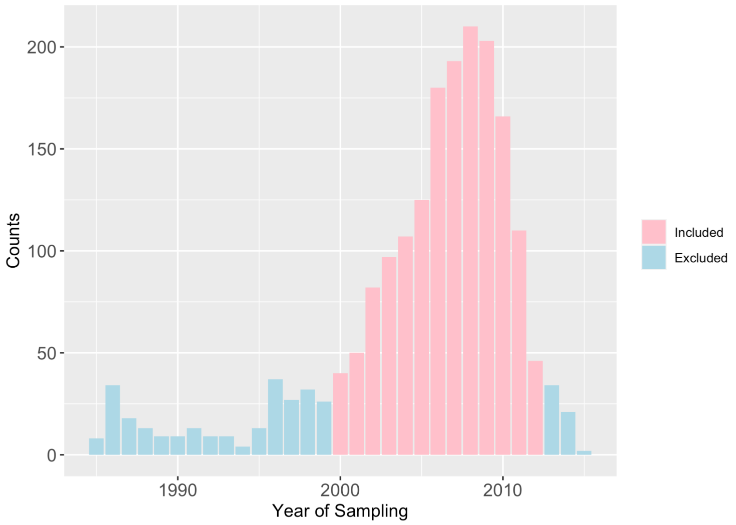

Supplementary Figure 1.

Distribution of year of sampling for all samples. Samples from 2000-2012 are included (colored in pink) in the Gaussian process regression analysis.

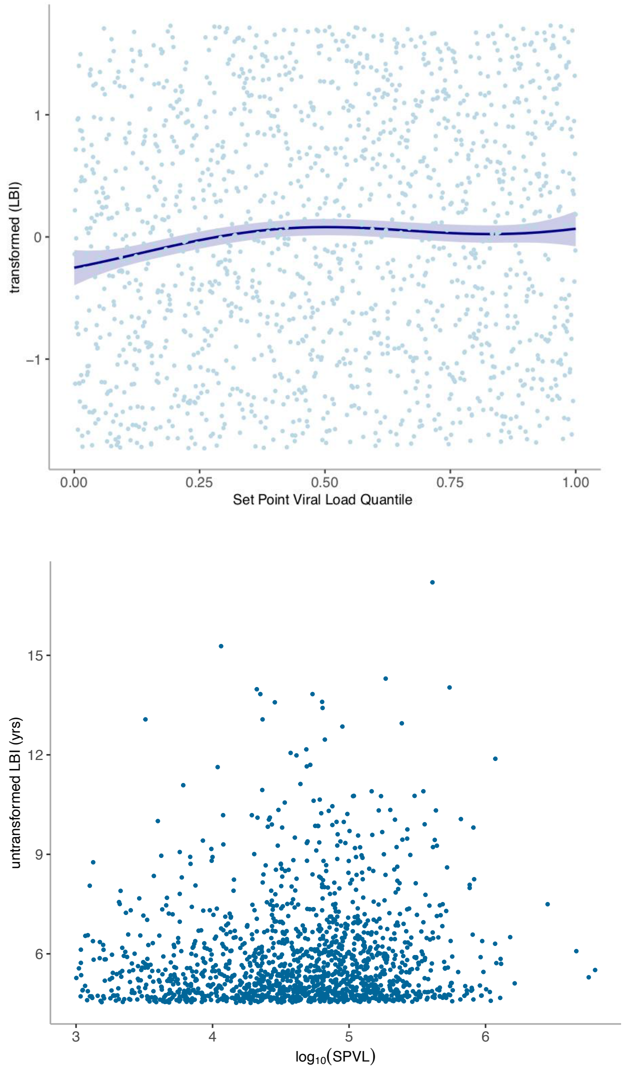

Supplementary Figure 2.

(top) Quantile spread of set-point viral load and corresponding transformed LBI values. Light blue points are data points in SPVL quantile and transformed LBI values, the blue line and shading are the posterior mean and 95% confidence interval from Gaussian process regression. (bottom) Untransformed LBI (yrs) values of the samples and their corresponding $\log_{10}$(SPVL) values.

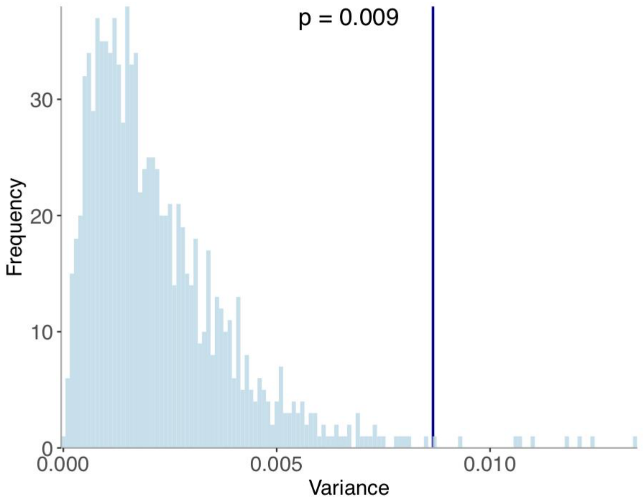

Supplementary Figure 3. Variance distribution of Gaussian process regression posterior mean permutations (N=1000). The dark blue line is the variance (σ^2 = 0.0087) of the non-permuted LBI vs. SPVL posterior mean curve.

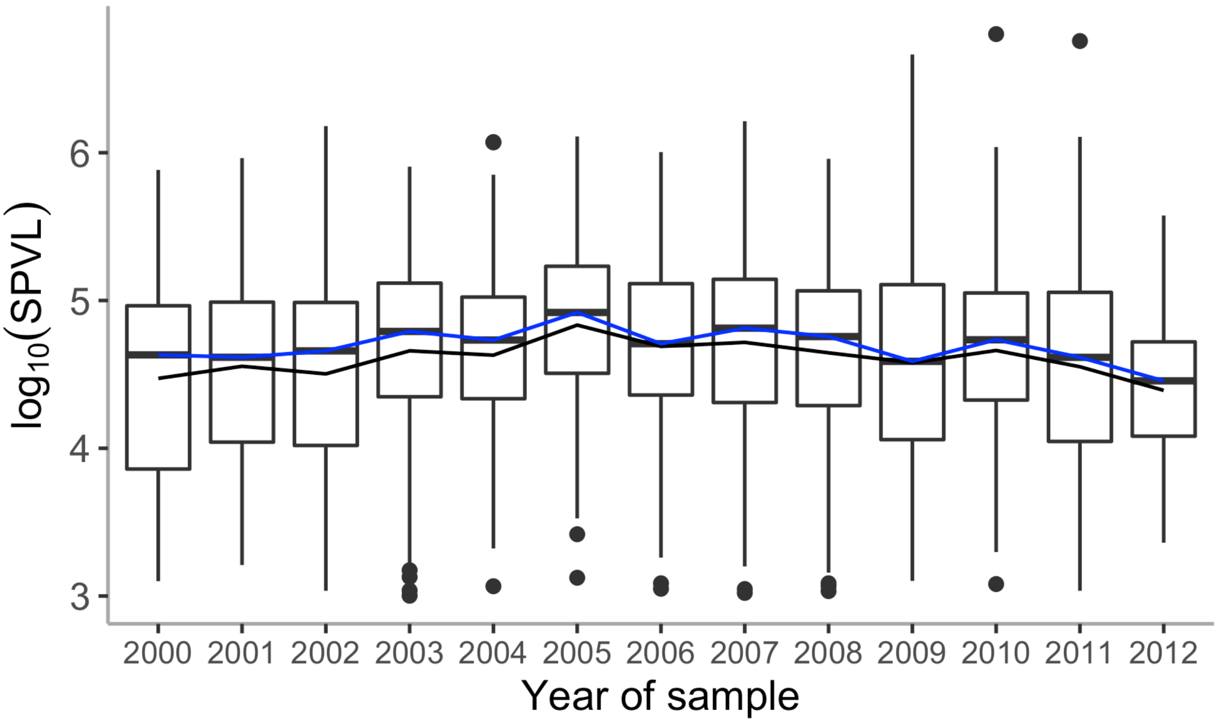

Supplementary Figure 4. Sample SPVL distribution per year (2000-2012). Black line connects mean SPVL among samples from each year, blue line connects median SPVL among samples from each year. Box hinges are the first and third quartiles, whiskers are not longer than 1.5 IQR and black dots are outliers beyond whiskers.

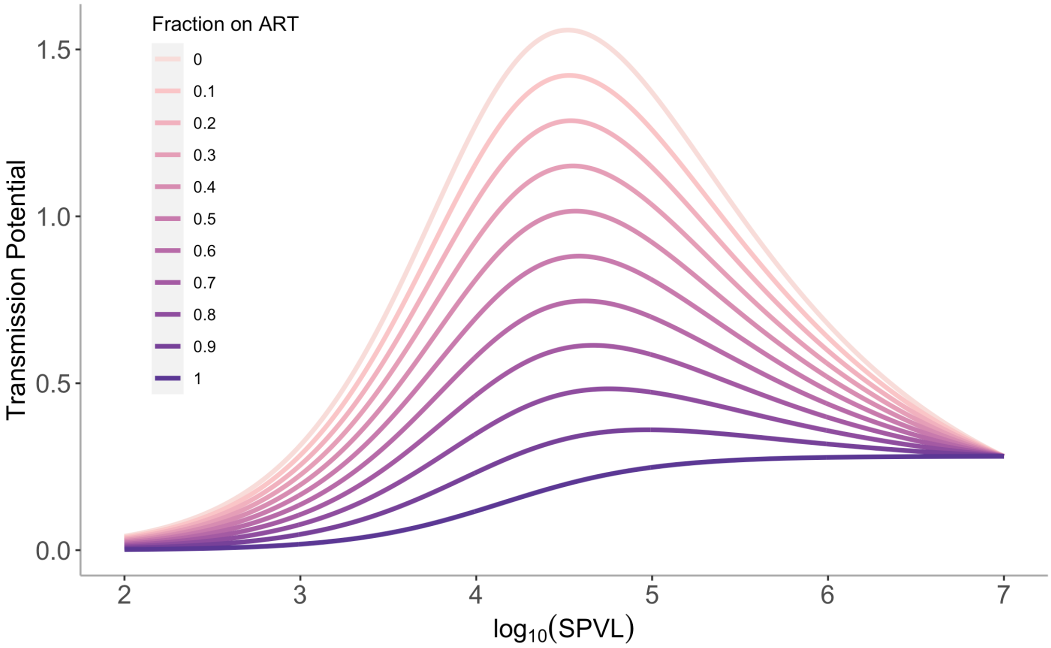

Supplementary Figure 5. The transmission potential as a function of SPVL for different values of the fraction of infected population given ART between two and four years from infection. Modelled assuming perfect ART, and infectiousness and duration of the infectious state functions from Fraser et al 2007.
