# Supplementary material for: Phylogenetic estimation of the viral fitness landscape of HIV-1 set-point viral load
Source: Virus Evol. 2022 Mar 16;8(1):veac022. doi: 10.1093/ve/veac022 (PMC8986633; doi:10.1093/ve/veac022)
Supplement: veac022_Supp [file veac022_supp.zip › Supplementary File 1.pdf]

Supplementary Information: Phylogenetic estimation of the viral fitness landscape of HIV-1 set-point viral load

Lele Zhao, Chris Wymant, François Blanquart, Tanya Golubchik, Astrid Gall, Margreet Bakker, Daniela Bezemer, Matthew Hall, Swee Hoe Ong, Jan Albert, Norbert Bannert, Jacques Fellay, M. Kate Grabowski, Barbara Gunsenheimer-Bartmeyer, Huldrych F. Günthard, Pia Kivelä, Roger D. Kouyos, Oliver Laeyendecker, Laurence Meyer, Kholoud Porter, Ard van Sighem, Marc van der Valk, Ben Berkhout, Paul Kellam, Marion Cornelissen, Peter Reiss, Christophe Fraser, Luca Ferretti, on behalf of the BEEHIVE Collaboration

Expected value of Local Branching Index for a birth-death-sampling process

We consider a birth-death (BD) or birth-death-sampling (BDS) model with fixed birth and death rates, but with sampling rate variable in time. We assume that all sampling events correspond to deaths, and that all tips of the tree correspond to sampling events. The model is fully described by the following parameters:

β = birth rate;

δ = death rate;

$s(t)$ = fraction of deaths sampled at time t before present;

T = time elapsed between the beginning of the birth-death process and the present time (assuming a single lineage at the beginning).

The Local Branching Index (LBI) of a point x on the tree is defined as the total weighted length of the tree, with weights exponentially decreasing with distance from x, as in the Main Text and in Neher et al (eLife 2014). The time scale used in the estimation of the LBI is denoted by τ, hence

$$LBI(x) = \int_{\text{tree}} dy \; e^{-d(x,y)/\tau} \tag{1}$$

General expression for the expected LBI

Given the Markov and interchangeability properties of BD/BDS models, all extant lineages at a given point in time are interchangeable and have the same distribution for their future evolution. Hence, the expected LBI depends only on time. Moreover, being a linear function of branch lengths, the expected LBI of a point x on the tree at height h before the present can be decomposed as

- the expected LBI for the future lineage(s) starting at x, given by $\nu l(h)$ where $l(h)$ is the expected forward-in-time LBI at height h, and $\nu = \begin{cases} 0 & \text{for a tip} \\ 1 & \text{for a lineage} \\ 2 & \text{for a node} \end{cases}$
- the expected LBI of the ancestral lineage leading to x, given by $\int_h^T dt \; e^{-(t-h)/\tau}$
- the expected LBI of all lineages branching from the ancestral lineage, given by $e^{-(t-h)/\tau} l(t)$ for an individual lineage branching at height t; the probability of such a lineage is βdt.

Overall, this leads to the general equation

$$\mathrm{E}[LBI|h] = \nu l(h) + \int_h^T dt e^{-(t-h)/\tau}(1 + \beta l(t)) \tag{2}$$

LBI for the full tree

The expected LBI of the full tree generated by the birth-death process, estimated for a position in the tree at height h since the present time, can be derived by summing all the contributions along the ancestral and future

lineage to that position as discussed above. This argument leads to the equation

$$\mathrm{E}[LBI|h] = \nu l_f(h) + \int_h^T dt e^{-(t-h)/\tau}(1+\beta l_f(t)) \tag{3}$$

The expected forward-in-time LBI $l_f(t)$ for the full tree satisfies the equation:

$$\frac{dl_f}{dt} = 1 + (\beta - \delta - 1/\tau) l_f(t) \quad , \quad l(0) = 0 \tag{4}$$

obtained by combining the contributions from new segments of tree ($+dt$), birth events ($+l(t)\beta dt$), death events ($-l(t)\delta dt$) and decay of LBI with tree distance ($-l(t)dt/\tau$). The above equation has the solution

$$l_f(t) = \frac{1 - e^{-(1/\tau - \beta + \delta)t}}{1/\tau - \beta + \delta} \tag{5}$$

The final solution for the LBI is

$$\begin{aligned}\mathrm{E}[LBI|h] =& \frac{1}{1/\tau - \beta + \delta}\Bigg[\ \nu\left(1 - e^{-(1/\tau-\beta+\delta)h}\right) + (1+\tau\delta)\left(1 - e^{-(T-h)/\tau}\right) + \\ & + \frac{\beta}{2/\tau - \beta + \delta}\left(e^{-(2/\tau-\beta+\delta)h} - e^{-(2/\tau-\beta+\delta)T}\right)\ \Bigg]\end{aligned} \tag{6}$$

Note that the above expression behaves very differently depending on the value of τ, with three different behaviours if $1/\tau > \beta - \delta$, $1/\tau < \beta - \delta < 2/\tau$ or $2/\tau < \beta - \delta$. The interpretation is most meaningful and interesting for the first one (i.e. small τ).

In fact, for small τ, the approximate value of LBI in the middle of a large epidemic, used in the Main Text, is

$$\mathrm{E}[LBI|h] = \frac{1 + \tau\delta}{1/\tau - (\beta - \delta)} \tag{7}$$

and corresponds to the limit $\tau \ll h \ll T$.

LBI restricted to the sampled subtree

The topology of the full tree can be very different from the topology of the subtree generated by all sampled tips. For simplicity, we assume a sampling

probability $s(h)$ per each death event occurring at time h before present, i.e. a rate of sampling $s(h)\delta$ and a rate of unsampled death $(1-s(h))\delta$.

The expected LBI of the full tree generated by the birth-death process, estimated for a position in the tree at height h since the present time, can be derived by an expression similar to the one above, with a slight twist due to the assumption that the position belongs to the sampled subtree:

$$\mathrm{E}[LBI|h] = \nu \frac{l_s(h)}{p_s(h)} + \int_h^T dt e^{-(T-h)/\tau}(1+\beta l_s(t)) \tag{8}$$

where $l_s(h)$ is the expected forward-in-time LBI of the *sampled* part of the lineage, $p_s(h)$ is the probability that there is at least a sampling event in a clade descending from a single lineage at time h before present. It is easy to show that the ratio $l_s(h)/p_s(h)$ is the expected forward-in-time LBI for the sampled component of the lineage *conditional* on the existence of a sampled component.

The equation for $l_s(t)$ can be easily derived as above, but considering that the contribution of a new segment of the tree is conditional on the lineage being sampled (hence $+p_s(t)dt$):

$$\frac{dl_s}{dt} = p_s(t) + (\beta - \delta - 1/\tau)l_s(t) \quad , \quad l_s(0) = 0 \tag{9}$$

On the other hand, the equation for $p_s(t+dt)$ combines some mutually exclusive probabilities:

- the probability of a new sampled tip, i.e. $\delta dt s(t)$;
- the probability that the only underlying lineage is sampled, i.e. $(1 - \beta dt - \delta dt)p_s(t)$;
- the probability that any of the two underlying lineages is sampled, i.e. $\beta dt[1-(1-p_s(t))^2]$.

The resulting differential equation is

$$\begin{aligned}\frac{dp_s}{dt} =& \delta s(t) + \beta[1-(1-p_s(t))^2] - (\beta+\delta)p_s(t) = \\ =& (\beta-\delta)p_s(t) - \beta p_s(t)^2 + s(t)\delta \quad , \quad p_s(0) = 0\end{aligned} \tag{10}$$

The system of equations (8),(9),(10) can be easily solved numerically for any choice of sampling fraction $s(t)$.
