# Supplementary material for: Phylogenetic estimation of the viral fitness landscape of HIV-1 set-point viral load
Source: Virus Evol. 2022 Mar 16;8(1):veac022. doi: 10.1093/ve/veac022 (PMC8986633; doi:10.1093/ve/veac022)
Supplement: veac022_Supp [file veac022_supp.zip › Supplementary File 2.pdf]

Supplementary File 2

Modelling transmission potential with Anti-Retroviral Treatment (ART)

We modelled transmission potential as a function of SPVL using the parameters from Fraser et al. 2007, while varying the fraction of infected individuals given ART at random times between 2-4 years from infection. The model assumes that ART prevents transmissions perfectly by reducing viral load to undetectable levels.

Equation (modified from Fraser et al. 2007):

$$Tp(V,f) = \int_2^4 dt\,\left[(1-f)D(V) + f\min\big(t, D(V)\big)\right]\beta(V)$$

$$D(V) = \frac{D_{max}{D_{50}}^{D_k}}{\left[V^{D_k} + {D_{50}}^{D_k}\right]},\ \beta(V) = \frac{\beta_{max}V^{\beta_k}}{\left[V^{\beta_k} + {\beta_{50}}^{\beta_k}\right]},$$

with D_{max} = 25.4 years, D_{50} = 3058 copies per mL peripheral blood, D_k = 0.41, β_{max} = 0.317 per year, β_{50} = 13938 copies per mL peripheral blood, β_k = 1.02.
